# Drying Characteristics and Physical and Nutritional Properties of Shrimp Meat as Affected by Different Traditional Drying Techniques

**DOI:** 10.1155/2016/7879097

**Published:** 2016-03-13

**Authors:** P. T. Akonor, H. Ofori, N. T. Dziedzoave, N. K. Kortei

**Affiliations:** ^1^Council for Scientific and Industrial Research-Food Research Institute, P.O. Box M 2O, Accra, Ghana; ^2^Graduate School of Nuclear and Allied Sciences, University of Ghana, P.O. Box LG 80, Legon, Ghana

## Abstract

The influence of different drying methods on physical and nutritional properties of shrimp meat was investigated in this study. Peeled shrimps were dried separately using an air-oven dryer and a tunnel solar dryer. The drying profile of shrimp meat was determined in the two drying systems by monitoring moisture loss over the drying period. Changes in color, proximate composition, and rehydration capacity were assessed. The rate of moisture removal during solar drying was faster than the air-oven drying. The development of red color during drying was comparable among the two methods, but solar-dried shrimps appeared darker (*L* ^⁎^ = 47.4) than the air-oven-dried (*L* ^⁎^ = 49.0). Chemical analysis indicated that protein and fat made up nearly 20% and 2% (wb) of the shrimp meat, respectively. Protein and ash content of shrimp meat dried under the two dryer types were comparable but fat was significantly (*p* < 0.05) higher in oven-dried meat (2.1%), compared to solar-dried meat (1.5%). Although rehydration behavior of shrimp from the two drying systems followed a similar pattern, solar-dried shrimp absorbed moisture more rapidly. The results have demonstrated that different approaches to drying may affect the physical and nutritional quality of shrimp meat differently.

## 1. Introduction

Shrimp belongs to a large group of crustaceans with extended abdomen and it is one of the most important commercial seafood in the world [1]. It is very popular in Ghana and fished on both commercial and artisanal scale around the Keta to Ada and Axim to Cape 3 Points areas [2]. Shrimps are estimated to contain nearly 20% protein (wb) with well-balanced amino acids and significantly high amounts of other nutrients including micronutrients such as calcium and selenium. Lipids in shrimps are largely made up of polyunsaturated fatty acids which are essential for human health [3, 4]. They have also been identified as rich in vitamin B12 and astaxanthin, a fat-soluble carotenoid which has antioxidant properties [5].

The high moisture content of shrimps, together with its high protein content, predisposes them to rapid deterioration. They begin to go bad shortly after capture, unless they are subjected to cold storage, a condition which extends its shelf life significantly. However this means of storage is expensive and may not be available in certain areas where electricity is a challenge. In Ghana and most parts of the world, drying remains one of the best options of preprocessing this seafood. It is one of the oldest means of food preservation and is applicable to a wide range of food products including shrimps. The principle behind drying is primarily reduction of moisture to levels low enough to prevent microbial growth and also slow down enzymatic and other biological reactions that may contribute to food spoilage. Dried shrimps are popular and widely acceptable. They are used (in whole or powdered form) in soups and sauces as a major protein source and for their delectable flavor.

Several drying techniques have been applied to process shrimps. Some of these methods are freeze-drying [6], super-heated steam drying [7], jet-spouted bed drying [8], and heat pump drying [9], among others. Notwithstanding these improved approaches to drying shrimps, traditional sun or solar drying and hot air drying remain the most widely applicable means of processing shrimps in Ghana and most developing countries. This is because the production of dried shrimps is mostly done on artisanal and small scales which require less sophistication and relatively cheaper throughputs. Solar and hot air drying are known to affect most food products but information on their effect on the nutritional and physical quality indices of shrimps is scanty.

The effects of the aforementioned traditional drying techniques on shrimps need to be elucidated because changes that occur as a result of drying are likely to affect the quality and, consequently, market value of the final product. The aim of the present study, therefore, was to determine the effect of solar tunnel drying and air-oven drying on drying characteristics and physical and nutritional properties of shrimp meat.

## 2. Materials and Methods

Fresh marine shrimps (*Penaeus notialis*) were procured from a fish market in Accra and transported on ice in a cold box to the laboratory. The shrimps were sorted and cleaned by washing in potable water before the head and shells were removed and discarded. The shrimp meat was then divided into two batches and subjected to different types of drying.

### 2.1. Drying Experiments

Drying experiments were conducted in two types of dryers; a mechanical air-oven dryer (Apex B35E, London) and a solar tunnel dryer (fabricated locally by the CSIR-Food Research Institute, Ghana). Shrimp meat, weighing 200 g (in triplicates), was spread in a single layer on a wire mesh and loaded into the convective hot air dryer or solar tunnel dryer. The samples were dried for 17 hrs at 55.0 ± 1.5°C in the mechanical dryer whereas solar drying was accomplished in 20 hrs at a mean temperature of 57.4 ± 6.9°C. Final moisture contents were 9.97 ± 0.33% and 9.68 ± 0.26% correspondingly for air-oven- and solar-dried shrimp meat.

Moisture loss during drying was determined by measuring the loss in weight of samples at hourly interval, with an electronic balance (Kern 510, Kern and Sohn, GMbH, Germany). Sampling and weighing were done until a fairly constant weight was attained [10]. The initial moisture content of shrimp meat, determined by standard methods [11], was 77.12 ± 0.06% (wb). Dried shrimp meat was sealed air-tight in flexible polypropylene bags and stored at room temperature.

### 2.2. Color Measurements

Color of the shrimp meat was measured with a Minolta tristimulus color meter (CR-310, Minolta, Japan), calibrated with a reference white porcelain tile (*L* = 97.63, *a* = 0.31, and *b* = 4.63). Measurements were done in triplicates and color described in *L* ^*∗*^, *a* ^*∗*^, and *b* ^*∗*^ notation, where *L* ^*∗*^ is a measure of lightness, *a* ^*∗*^ defines components on the red-green axis, and *b* ^*∗*^ defines components on the yellow-blue axis. Color determinations were done at hourly intervals over a period of 8 hrs for both mechanical and solar-dried shrimp meat.

### 2.3. Proximate Composition

Shrimp meat was analyzed for moisture, ash, crude fat, and crude protein using approved methods 925.10, 920.87, 920.85, and 923.03 of the Association of Official Analytical Chemists [11]. Determinations were carried out before and after drying by the two methods.

### 2.4. Rehydration Studies

Dried shrimp meat was rehydrated according to the method by Doymaz and Smail [12]. Five grams of dried shrimp meat was rehydrated in distilled water at room temperature using a sample to water ratio of 1 : 40. At 30 min interval, shrimp meat was removed and carefully blotted with tissue paper, weighed on an electronic balance, and immediately returned into the same soaking water. Dried shrimps were rehydrated over a period of 5 hrs when the weight of rehydrated samples had stabilized. Rehydration ratio was then calculated using the following relation [13]:

(1) $$\begin{array}{c} \text{Rehydration Ratio}=\frac{\text{Mass of rehydrated sample}}{\text{Mass of dried sample}}. \end{array}$$

## 3. Results and Discussion

### 3.1. Drying Profile

**Figure 1:**
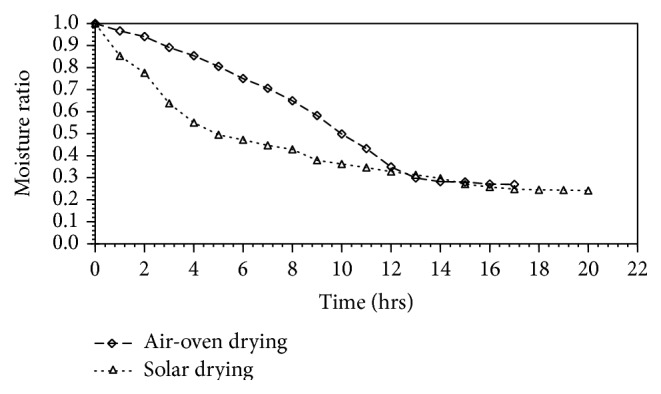
Variation of moisture ratio in solar- and air-oven-dried shrimp meat.

The drying curves (Figure 1) show a faster rate of moisture removal during solar drying than air-oven drying, especially within the first 12 hours of drying. This outcome may be attributed to the higher average drying temperature (57°C) and possibly air speed, in the solar dryer compared to the air-oven (55°C) which is thermostat controlled. This, together with higher fluctuation in solar drying temperature (SD of ±6.9), may also explain the variation in arriving at a stable weight during drying. A stable weight was reached faster in air-oven compared to solar drying.

In all two drying methods, a decline in the rate of moisture removal was observed after 12 hrs of drying until a fair stability in moisture content was established. This may be explained by the difficulty in moisture removal as the drying front recedes towards the innermost parts of the shrimp meat and resistance to moisture movement becomes higher [14].

### 3.2. Proximate Composition

Proximate composition of shrimp meat before and after drying indicates an interesting nutrient profile. As is typical of most sea foods, the fresh shrimp was made up of nearly 80% moisture. This result compares well with 80.5% and 77.2% reported in earlier studies for black tiger shrimp and white shrimp, respectively [4]. After drying, using the two methods, moisture was reduced to about 10%, which is less than the specified moisture for dried shrimps [15]. Low moisture content in dried shrimps is encouraged to safeguard the product from microbial attack and enzymatic action and therefore prevent spoilage. Moisture of shrimp from the two drying methods did not show any significant differences (*p* > 0.05), although air-dried shrimp meat appeared to have a lower moisture content.

After moisture, protein was the second most abundant component of the shrimp meat, and this made up about 86% (db) of fresh meat (Table 1). Shrimp meat from this study was slightly richer in protein compared to both black tiger and white shrimps reported by Sriket et al. [4], but lower than white leg shrimp [16] and for green tiger and speckled shrimps [3]. The discrepancies in protein content of shrimps used in these studies may be attributed to differences in species, growth stage, season, and waters in which they were shrimped [17]. The high protein content of shrimp meat makes it a good source of amino acids for human diets. Protein content of air-oven-dried shrimp meat was slightly lower but not significantly different (*p* > 0.05) from solar-dried shrimp meat. The difference in protein content before and after drying, which was remarkable (*p* < 0.05), may be related to protein denaturation and or the incidence of browning reactions, in which some amount of amino acid is used up.

Sriket et al. [4] have reported in previous studies that the fat content of some shrimp species ranges between 1.2 and 1.3% (wb). Most of this exists as membrane lipids as in the case of fish muscles [4, 18]. In the present study, shrimp meat contained about 2% fat (wb), but this reduced significantly (*p* < 0.05) to less than 2% after drying. This reduction suggests that, during drying, fat may have exuded along with moisture evaporation or oxidized into other compounds [19] since shrimp lipids are mainly made up of polyunsaturated fatty acids. Degradation of astaxanthin in the process of drying may also have contributed to the loss of lipids since this carotenoid is thought to have a high antioxidant activity [20]. The loss of fat was markedly different (*p* < 0.05) for the two drying methods, with solar-dried shrimps being the most severe. This observation is ascribed to higher drying temperature in the tunnel solar dryer. Similar results of fat loss as a result of drying were obtained by Wu and Mao [19] for dried grass carp fillets.

Ash represents the total mineral content in food and is essential in maintaining several bodily functions. Shrimp meat was found to contain appreciable amounts of ash, totaling nearly 1.6% (wb). This makes shrimp meat a good source of minerals in the diet. Due to moisture loss and concentration of chemical components, higher ash content was obtained after drying. This seemingly increased ash content was however not significantly different (*p* > 0.05) among the two drying methods. The ash content of shrimp meat in the present study is slightly higher than the ranges of 1–1.5% and 1.47% independently reported by Gunalan et al. [16] and Yanar and Çelik [3].

### 3.3. Color Development

**Figure 2:**
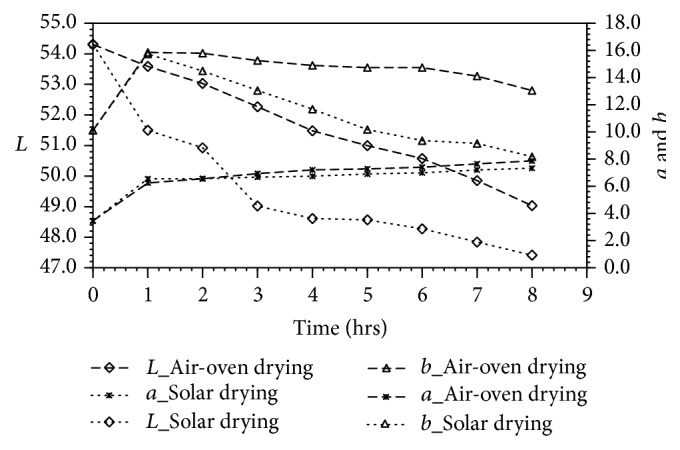
Color profile of shrimp meat during drying.

According to Yanar et al., [21], the market value of shrimp is dependent on the visual appearance of their body color, and this is attributed to the presence of the astaxanthin. This carotenoid pigment is responsible for the red-orange tissue pigmentation of shrimp meat.  Figure 2 shows color development in shrimp meat as monitored over the drying period.

Lightness index (*L* ^*∗*^) reduced with the passage of time, suggesting that shrimp meat became darker. Darkening may have occurred because of Maillard browning reactions which took place during drying. The extent of these reactions in solar drying may have been more pronounced, resulting in darker shrimp meat compared to oven-dried samples. Again, the results show that red color (*a* ^*∗*^) was developed when shrimp meat was dried, and the two drying methods showed similar (*p* > 0.05) results. Development of redness on exposure of shrimp meat to heat is as a result of the release of astaxanthin when carotenoproteins breakdown during protein denaturation. In both solar and air-oven drying, the intensity of redness increased nearly twofold within the 1st hour of drying and only increased slightly thereafter. This is attributable to an increase in concentration of astaxanthin when water was removed from shrimp tissue [8]. Yellowness (*b* ^*∗*^) of shrimp meat also increased over the 1st hour of drying as a result of formation of yellow pigments from browning reactions. However, a steady decline in yellowness (*b* ^*∗*^) was noticed after 1 hour of drying. The dip in yellowness was marginal in air-oven drying but more severe in solar drying as in the case of lightness index (*L* ^*∗*^).

### 3.4. Rehydration

**Figure 3:**
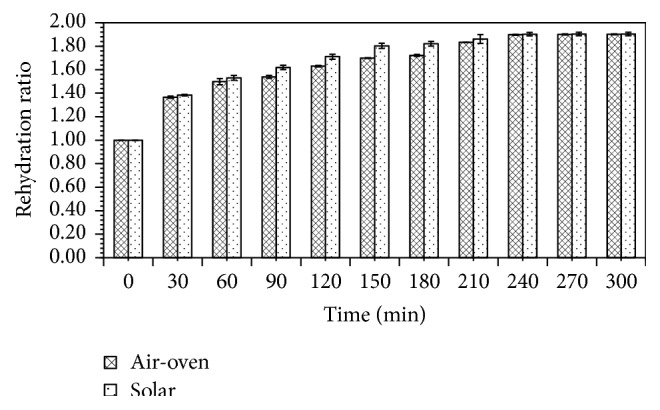
Rehydration characteristics of dried shrimp meat.

Rehydration refers to the process of moistening a dried product and is an indicator of quality criterion in most dried foods. It is an indicator of cellular and structural disintegration that occurs during dehydration [22]. Rehydration ratio of shrimp meat as a function of time is presented in Figure 3.

In agreement with earlier studies [10, 23, 24], there was a rapid increase in weight of shrimp meat from both air-oven and solar drying because of high rate of water absorption at the initial stages. The first 3 hours saw a rapid weight gain by the meat (from both drying methods) and this slowed down afterwards and, subsequently, flattened off between the 4th and 5th hours as the process reached equilibrium. The initial rapid uptake of moisture by shrimp meat, as posited by Sagar and Suresh Kumar [25], is the result of surface and capillary suction.

A comparison of the rehydration behavior of shrimp meat from the two drying systems indicated a clear difference, especially, between the 1st and 3rd hours of rehydration. Rehydration was faster in solar compared to mechanical drying. The extent and rate of water uptake during rehydration is largely influenced by cellular and structural arrangements in the food matrix since this provides the channels for conveying water to muscle fibers. This phenomenon has been amply demonstrated in previous studies [8, 26]. Also, as noted by Niamnuy et al. [8], higher protein contraction may have reduced the rehydration ability of mechanically dried shrimp meat.

## 4. Conclusion

The study showed differences in some chemical and quality properties between dried shrimps, using different techniques. Protein content generally remained unaffected by drying method, but the amount of fat was remarkably higher for oven-dried shrimp meat. Solar drying resulted in relatively darker shrimp meat with a higher rehydration rate, compared to oven-dried shrimp meat. Although drying occurred faster during solar drying, dried meat was of a lower quality compared to air-dried shrimp meat.

## Figures and Tables

**Table 1:** Chemical properties of fresh and dried shrimp meat (db).

Parameter	Fresh	Air-oven-dried	Solar-dried
Protein	86.21 ± 0.08^b^	85.64 ± 0.26^a^	84.89 ± 0.51^a^
Ash	6.93 ± 0.11^a^	6.82 ± 0.10^a^	6.77 ± 0.28^a^
Fat	6.54 ± 0.20^c^	5.98 ± 0.15^b^	5.74 ± 0.11^a^

Means with different superscripts along the same row are significantly different (*p* < 0.05).
